# Isolated cranial nerve disorder as presenting sign in multiple sclerosis: optic nerve versus “the others”

**DOI:** 10.3389/fneur.2025.1557326

**Published:** 2025-04-02

**Authors:** Arianna Di Stadio, Pietro Scribani Rossi, Diego Kaski, Chiara Zilli, Massimo Ralli, Evanthia Bernitsas, Marta Altieri

**Affiliations:** ^1^Department of Mental and Physical Health and Preventive Medicine, Otolaryngology Unit, University of Campania “Luigi Vanvitelli”, Napoli, Campania, Italy; ^2^Santa Lucia Hospital IRCSS, Rome, Italy; ^3^Neurology Department, University La Sapienza, Rome, Italy; ^4^Department of Clinical and Movement Neurosciences, UCL Queen Square Neurology, London, United Kingdom; ^5^Department of Health Sciences, UniCamillus - Saint Camillus International Medical University, Rome, Italy; ^6^Multiple Sclerosis Center, Department of Neurology, Wayne State University, Detroit, MI, United States; ^7^Multiple Sclerosis Center, Department of Neurology, University La Sapienza, Rome, Italy

**Keywords:** cranial nerve, optic nerve, optic neuritis, facial palsy, trigeminal pain, multiple sclerosis

## Abstract

**Background:**

The prevalence of cranial nerve involvement in Multiple Sclerosis (MS) varies across studies. It has been speculated that first presentation of disease with cranial nerve involvement – except for optic neuritis – may be associated with milder progression.

**Aim:**

This study compares the clinical outcome of patients with MS in a 4-year follow-up of patients with initial symptoms of optic neuritis (ON) versus those with other cranial nerve (OCN) involvement.

**Materials and methods:**

Retrospective analysis of MS patient database of a tertiary referral university MS center. We included treatment-naïve patients diagnosed with MS according to the revised McDonald criteria, who presented with their first clinical symptoms suggestive of ON or OCN. Patients were required to have regular clinical and radiological follow-up visits (at least two outpatient visits per year and one annual 1.5T MRI), and no comorbidities. The number of relapses and the Expanded Disability Status Scale (EDSS) scores were assessed at six-month intervals during clinic visits. The primary outcome was the number of relapses observed during the study period, comparing the ON and OCN groups. Several statistical analyses were performed, including multiple linear regression, Cox proportional hazards model, one-way ANOVA, and odds ratios, to compare the groups.

**Results:**

Of the 84 patients included, none had comorbities (e.g., overlap with other inflammatory diseases, neoplasm etc.). Fifty-five presented with ON and 29 with OCN (e.g., diplopia, trigeminal pain, hearing or vestibular symptoms) at onset. Patients with ON were younger than those with OCN symptoms (*p =* 0.02), had a higher risk of relapse (more than two relapses) (OR: 1.53) and greater disability (incremental EDSS) over the 4-year follow-up (OR: 1.60).

**Conclusion:**

Patients with OCN involvement at the onset experienced fewer relapses and had better EDSS scores at the 4-year follow-up compared to those with ON at onset. These preliminary findings suggest that MS onset with OCN involvement may be associated with a more favorable disease course.

## Introduction

The presenting features of multiple sclerosis (MS) vary across individuals, reflecting the widespread distribution of lesion in the supratentorial area, brainstem, and spinal cord (1, 2). While involvement of the optic nerves is highly predictive of MS in younger individuals, isolated cranial nerve involvement outside the optic nerves is less common and may lead to a delayed diagnosis (3, 4). In fact, only 7–10% of patients with MS (PwMS) present with isolated cranial nerve involvement (excluding optic neuritis) as the presenting sign (5–7). As a result, patients with optic neuritis (ON) are typically seen by either an ophthalmologist or neurologist, receiving prompt investigation, diagnosis, and treatment (2, 8). In contrast, patients whose presenting symptoms involve other cranial nerves (OCN) may be referred to non-neurology specialties (e.g., otolaryngology, dentistry, or audiovestibular medicine), who may be less familiar with MS, leading to a potential misdiagnosis and a subsequent delay in MS diagnosis (3, 9).

Recent studies have highlighted that symptoms such as auditory impairment, which are typically attributed to peripheral (middle or inner) ear conditions, can be indicative of a first presentation or relapse of MS (10–13). These symptoms may mimic peripheral cranial nerve involvement but, in fact, arise from a central nervous system lesion (10, 11).

It remains unclear whether isolated cranial nerve involvement as a presenting feature of MS can predict longer-term outcomes. The aim of this study was to evaluate, in a 4-year follow-up period, the clinical outcomes of PwMS who presented with optic neuritis versus those with other cranial nerve involvement.

## Materials and methods

This retrospective study included patients recruited from an outpatient database at a tertiary referral MS University center. The database from which the data were extracted was the same as that used in a previously published study (14). The study was approved by the Institutional Review Board (IRB) of the institution in January 2023. The database contained all patients who had been under active review at the MS center for at least 4 years at the time of this study.

Inclusion criteria for the study were as follows: pharmacotherapy naïve patients with diagnosis of MS according to revised 2017 McDonald criteria; patients with their first clinical symptom referable to ON or OCN (diplopia, trigeminal pain, facial palsy, hearing or vestibular symptoms); regular clinical and radiological follow-up visits (at least two outpatient visits per year and one annual 1.5T MRI); and the absence of comorbidity (e.g., overlap with other inflammatory diseases, neoplasms). Relapses and Expanded Disability Status Scale (EDSS) scores were assessed at 6-month intervals during clinic visits.

Demographic and clinical data were collected for each subject, including age, sex, age at onset, type of MS, and current therapies. All patients underwent annual brain and spinal MRI (1.5T). Three clinical and radiological outcome measures were used: (1) clinical relapses; (2) changes in EDSS scores; (3) MRI activity, defined as the absence of new or enlarged lesions on T2-weighted images and the presence of new gadolinium-enhancing (Gd+) lesions.

Exclusion criteria included: patients with brain (cerebrum) or spinal cord onset, patients who suffered from simultaneous optic nerve and other cranial nerve dysfunction at onset, patient with missing data at the 4-year follow-up, active smokers [due to an increased risk of relapses (15)], and those with other comorbidities (such as stroke, hypertension, cancer, or other neuroinflammatory or neurological diseases).

A relapse was defined as the appearance of new symptoms or signs that lasted for more than 24 h without concurrent fever or illness. Relapses were recorded by the treating physician during the face-to-face biannual visits. Relapses and EDSS scores were assessed at 6-month intervals during clinic visits. EDSS reassessments at each interval were conducted by the same physician with decennial experience (MA) to minimize intra-operator variability and reduce potential evaluation bias. All patients underwent annual brain and spinal MRI (1.5T). Data were collected from medical records and outpatient visits. If a relapse occurred between observation intervals, patients were re-evaluated with EDSS assessment and MRI.

MRI changes were evaluated by counting the number of lesions and assessing their location in the baseline MRI (first performed) compared to the follow-up findings. The presence of new enhancing and non-enhancing lesions was also recorded at the time of the MRI.

Based on the nerve involved at disease onset, patients were classified into either the optic nerve (ON) group or the other cranial nerve (OCN) group (III, IV, V, VI, VII, or VIII). None of the patients exhibited involvement of cranial nerves IX-XII.

### Statistical analysis

Two-tailed t-test (*τ*) was used to compare the age of the onset between ON and OCN groups. The EDSS of ON and OCN at the baseline and after 4-year follow-up were compared using one-way ANOVA and Bonferroni- Holmes (BH) *ad hoc* test. Two-tailed t-test (τ) was used to compare the months of relapse between ON and OCN group. Odds Ratio (OR) was used to compare the risk of relapsing between ON and OCN, the risk of progressing to a second-line treatment or alternative therapy, the risk of more than two relapses in the 4 years, and active lesions on MRI between ON patients and OCN. Chi-square (*χ*) was used to compare nominal data as sex distribution between the two groups and relapses. To evaluate the effect of multiple factors (gender, age, nerve involvement, number of new lesions, treatment) on the relapsing we performed multilinear regression and Cox Proportional Hazard model to complete the analyses. *p* was considered significant <0.05. All tests were performed using Stata®.

## Results

Out of the total dataset of 215 patients, 119 were excluded because the presenting symptom did not involve cranial nerves alone. From the remaining 96 patients, 10 were excluded due to missing data. Two patients who had a combination of optic neuritis (ON) and other cranial nerve involvement (OCN) at onset were also excluded; a total of 84 patients were included in the final statistical analyses. None of the 84 patients were smokers or former smokers.

**Figure 1:**
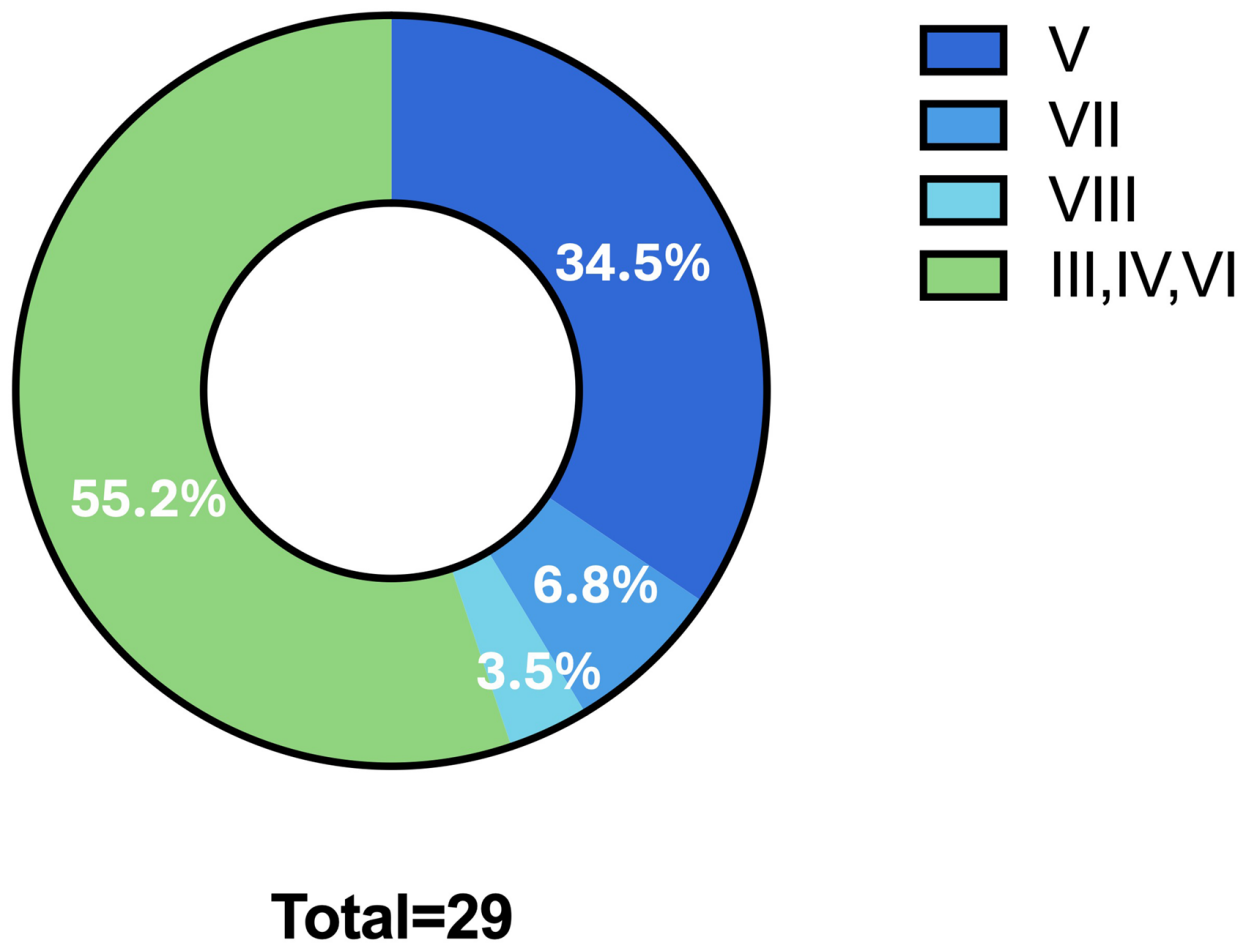
Prevalence of cranial nerve involvement.

Among these patients, 55 presented with ON as the initial symptom (65.5%), while the remaining 29 (34.5%) had OCN involvement (III, IV, V, VI, VII or VIII) as the initial symptom of MS (Figure 1). Of the 16 patients with ocular motor involvement, three had cranial nerve III affected (18.75%), 1 had cranial nerve IV involvement (6.25%), and the remaining 12 had cranial nerve VI involvement (75%).

The ON group included 16 males (29.1%) and 39 females (70.9%), while the OCN group included 10 males (35.5%) and 19 females (65.5%). The two groups were equally distributed by gender (*χ*: *p* = 0.90; *p* > 0.05). The average age at the onset was 31 ± 9.5 in the ON group and 36 ± 11.6 in the OCN group, with a statistically significant difference (*τ*: *p* = 0.02; *p* < 0.05) (Table 1).

**Table 1:** Demographic characteristics of the two groups at the baseline (top of the table) and at the end of the follow-up (bottom).

Group	Age	Women (*n*, %)	Men (*n*, %)	Baseline EDSS^*^
Optic nerve (ON)	31 ± 9.5	40 (71.4)	16 (28.6)	1.5 ± 1.1
Other cranial nerves (OCN)	36 ± 11.6	20 (55.5)	10 (45.5)	1 ± 0.3
*p* value	< 0.05	> 0.05	> 0.05	> 0.05

Group	Relapsing 1 time	Relapsing more than 1	New MRI lesions	Shifting therapy from first to second line	EDSS 4-years follow up*
Optic Nerve (ON)	27 (49%)	20 (36.4%)	27 (49%)	24 (43.6%)	2.1 ± 1.5
Other Cranial Nerves (OCN)	11 (37.9%)	7 (24.1%)	13 (44.8%)	8 (27.6%)	1.5 ± 0.8
*p* value	< 0.05	< 0.05	> 0.05	> 0.05	> 0.05

^*^Average and standard deviation.

**Figure 2:**
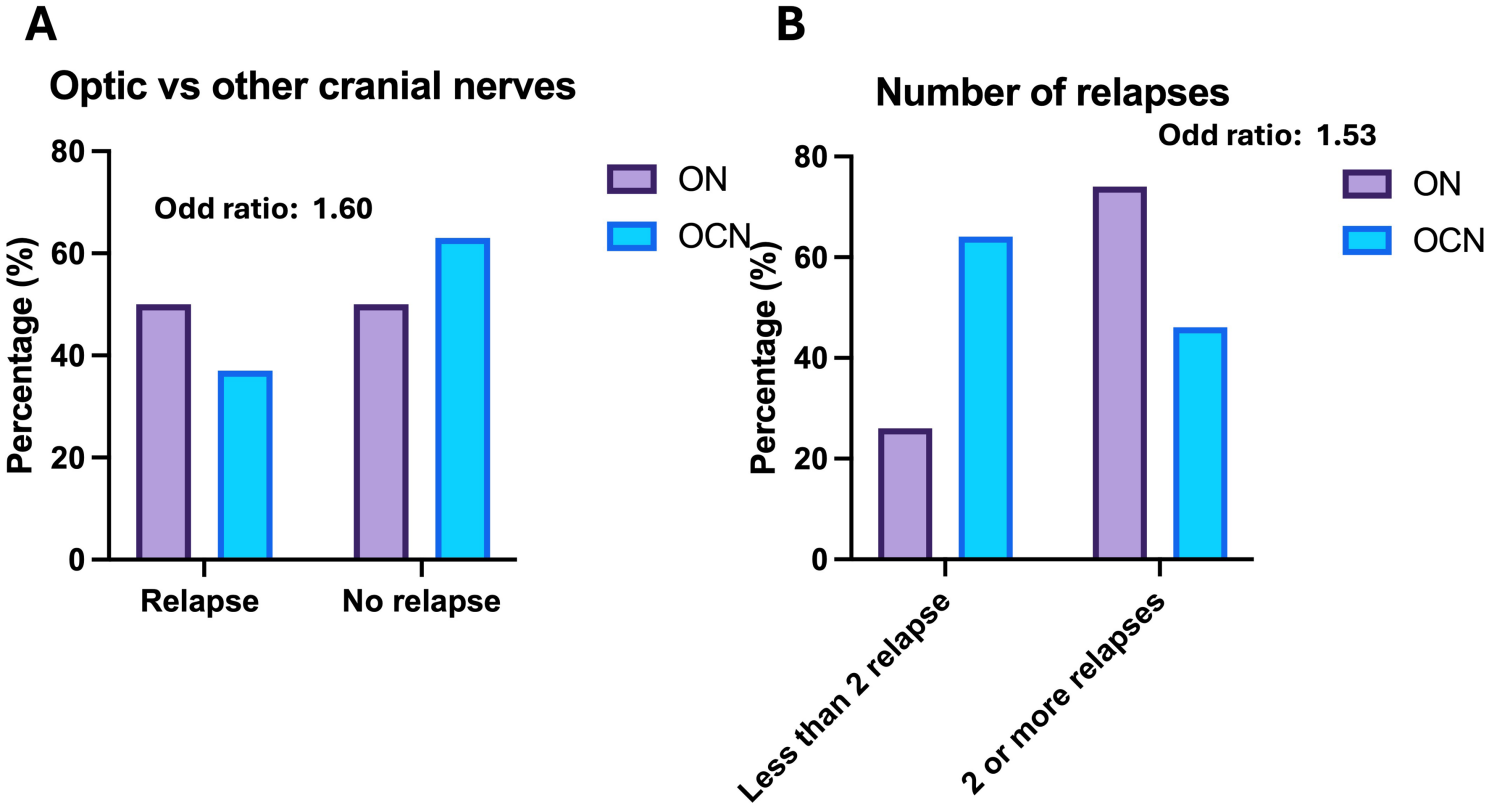
**(A)** Relapse versus no relapse in the 4-year follow-up in the groups. **(B)** Number of relapses during the observation period.

Twenty-seven patients in the ON group (49%) experienced at least one relapse during the follow-up, compared to 11 patients in the OCN group (37.9%). Patients in the ON group had a higher risk of relapse compared to those in the OCN group (OR: 1.60; CI 95%: 0.64–3.99; *p* = 0.04; *p* < 0.05) during the 4-year follow-up (Figure 2A). Of the 27 patients in the ON group, 20 (74%) had more than two relapses during the follow-up period, whereas 7 patients (63.6%) in the OCN group had more than two relapses. Patients with ON had an increased risk of experiencing more than two relapses over the 4-year period compared to the OCN group (OR: 1.53 CI95%: 0.83–2.83; *p* = 0.04 *p* < 0.05) (Figure 2B).

**Figure 3:**
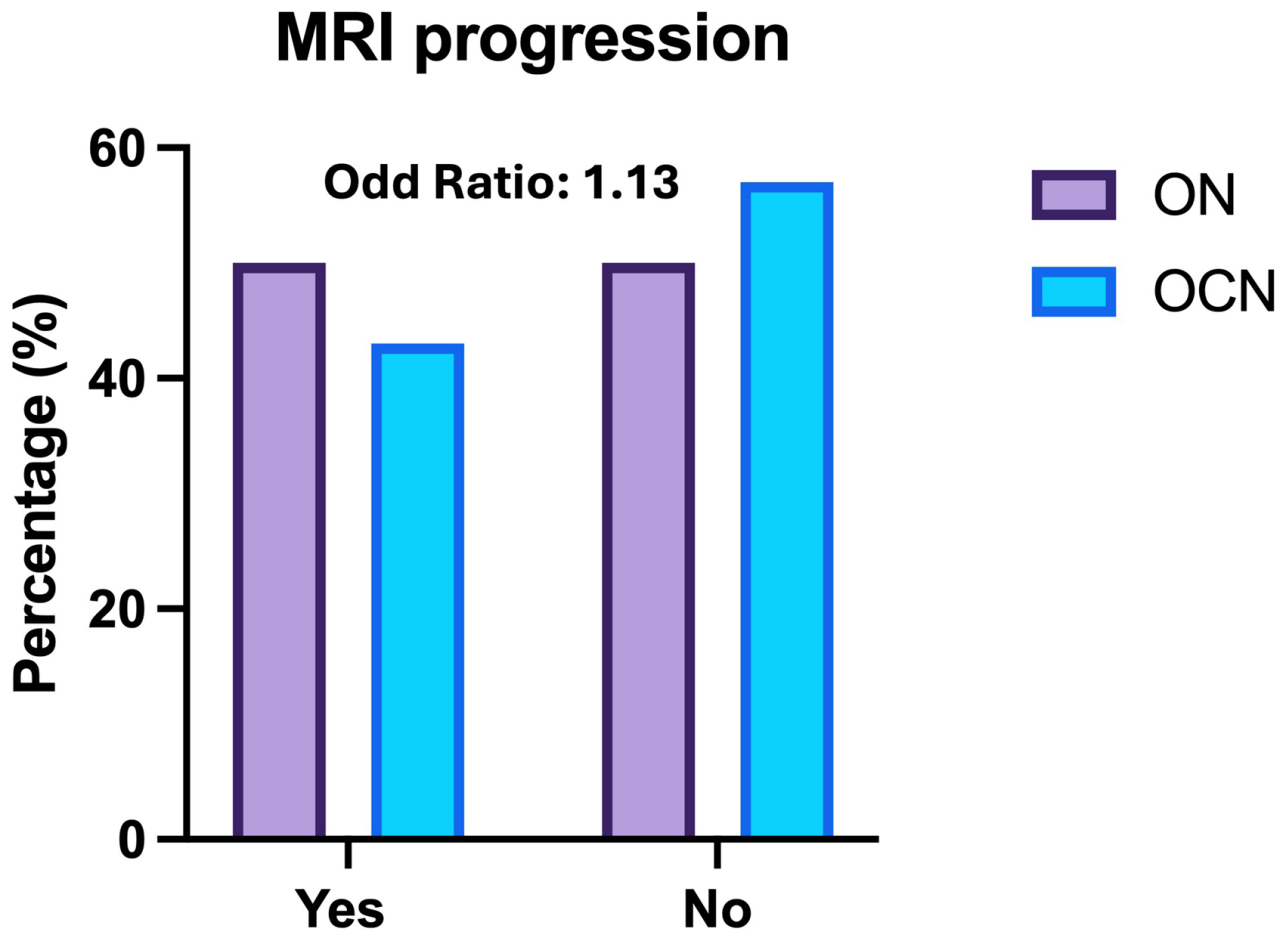
Histogram of radiological relapses (MRI) across the optic nerve (ON) and other cranial nerve (OCN) groups, showing no significant difference in the risk of new MRI lesions at 4-year follow up.

New MRI lesions were observed in 27 patients (49%) in the ON group and in 13 patients (44.8%) in the OCN group (OR: 1.13; CI95%: 0.46–2.76; *p* = 0.70; *p* > 0.05) over the 4-year follow-up period, with no statically significant difference (Figure 3). Three patients had new lesions in the spinal cord; two in the ON group (3.6%) and one in the OCN group (3.4%).

Twenty percent of patients (11 subjects) in the ON group and 34.4% of patients (10 subjects) in the OCN group had enhancing MRI lesions during the follow-up radiological investigation.

**Figure 4:**
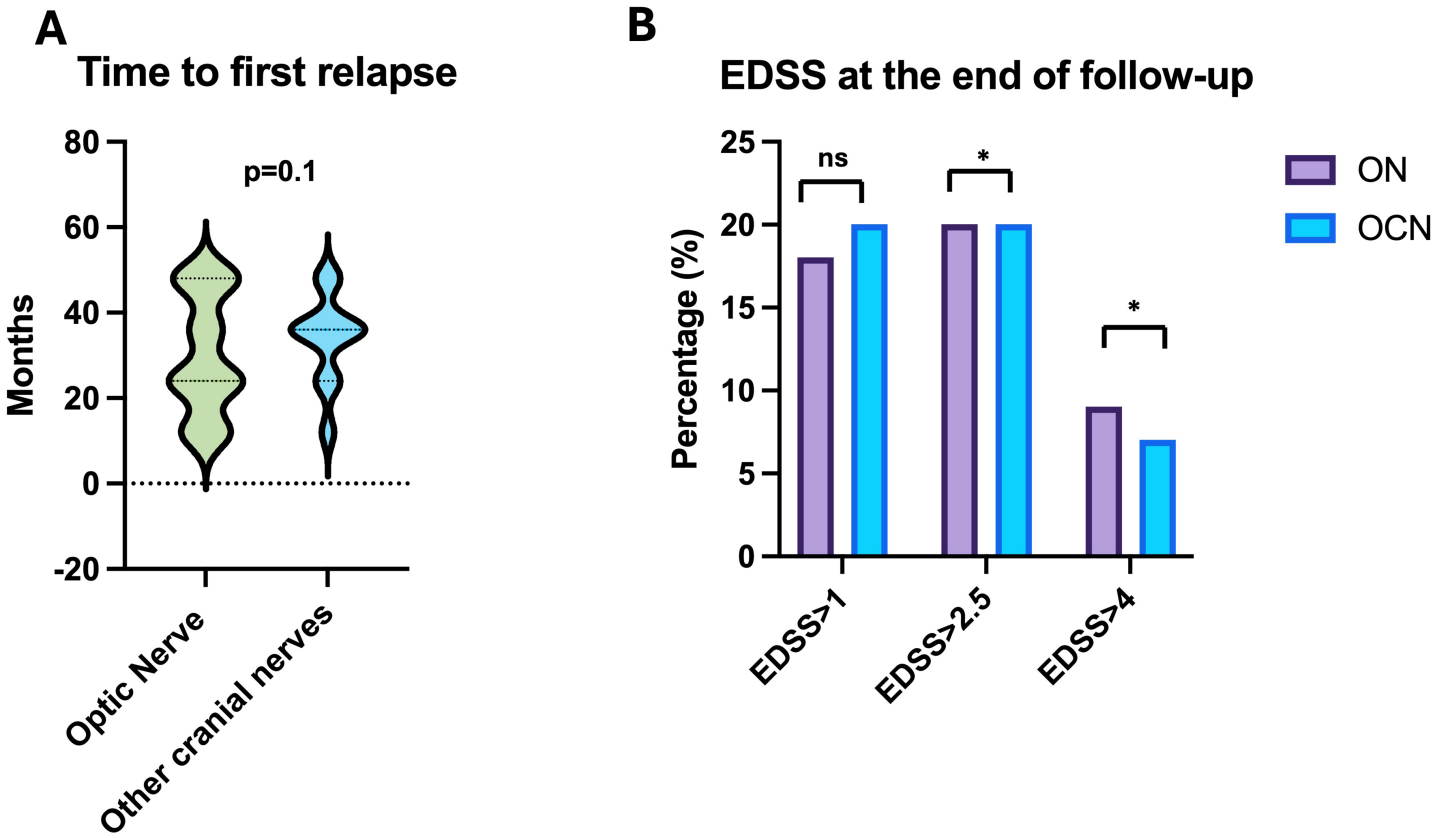
**(A)** Time to first relapse in ON and OCN groups; no statistically significant differences were found. **(B)** Patients in the ON group had worse EDSS scores, with more patients showing scores greater than 2.5 at the end of the follow-up period, with statistically significant *p*. *p < 0.05.

The average time to the first relapse was 30.2 ± 13.8 months in the ON group and 33.8 ± 10.5 months in the OCN group, with no statically significant difference (*τ*: *p* = 0.1; *p* > 0.05) (Figure 4A).

**Figure 5:**
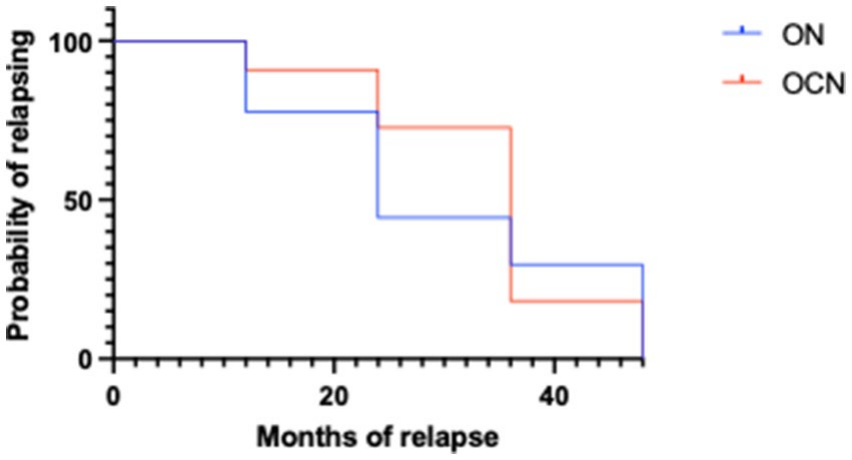
Survival curve in ON and OCN groups.

The Kaplan Meyer showed statistically significant differences between ON and OCN patients both for the likelihood of relapsing and the months of relapsing as illustrated in the survival curve, with strongly statistically significant difference (Figure 5) (*p* < 0.001).

Patients in the ON group showed a statistically significant increase in EDSS scores (clinical deterioration) (*τ*: *p* = 0.02; *p* < 0.05) when comparing the EDSS at symptom onset (average 1.5 ± 1.1 CI95%: 0–7) to the last follow-up (average 2.1 ± 1.5 CI95%: 0–7). In contrast, the EDSS in the OCN group did not show a statistically significant difference (τ: *p* = 0.10; *p* > 0.05) between baseline (average 1 ± 0.3 CI95%: 0–1.5) and the 4-year follow up (average 1.5 ± 0.8 CI95%: 0–2.5; Figure 4B).

Specifically, in the ON group, 10 patients (18.8%) scored more than 1, 11 patients (20.0%) scored more than 2.5, and 5 patients (9%) scored more than 4.5. In the OCN group, 6 patients (20.6%) scored more than 1, 6 patients (20.6%) scored more than 2.5, and 2 patients (6.8%) scored more than 4.5 (Figure 4B).

**Figure 6:**
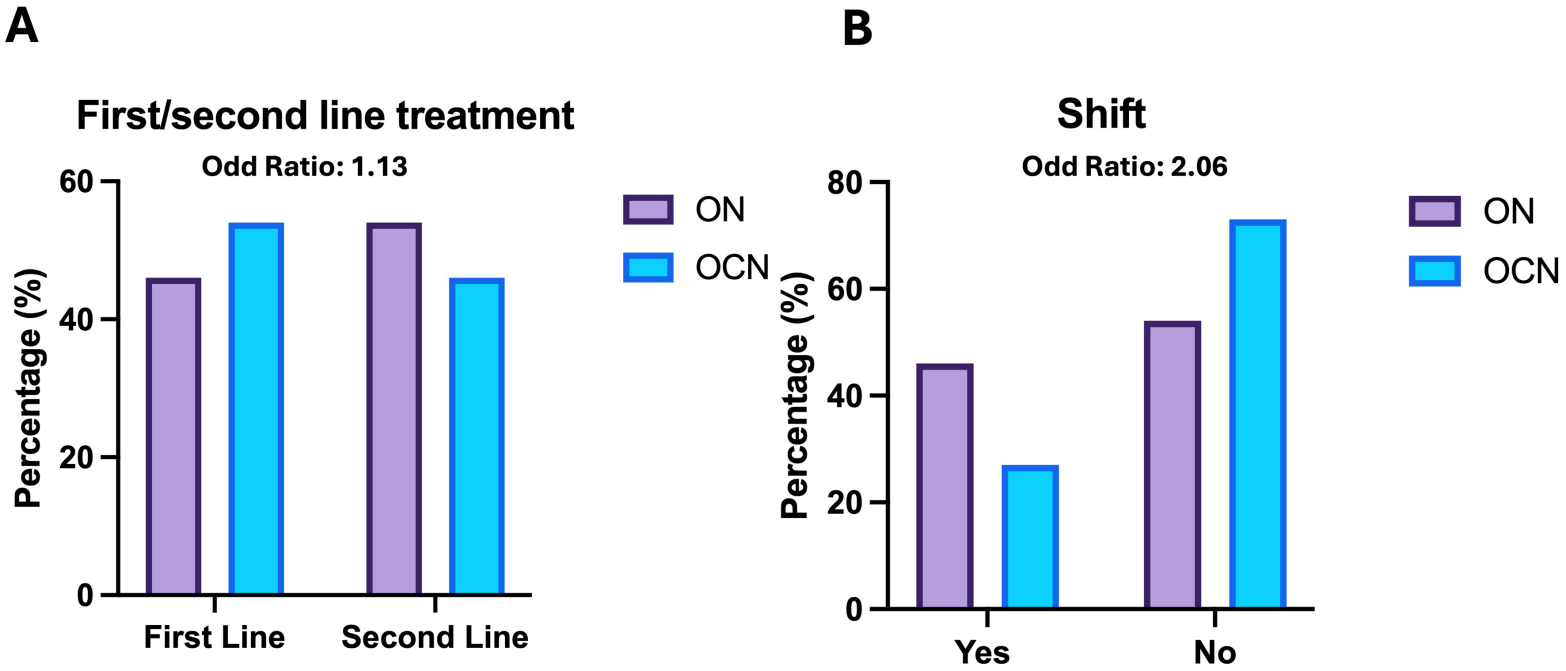
Therapies **(A)** and shift **(B)** in ON and OCN groups. While no significant differences were found in the use of first line or second line treatment, ON patients who started with first line treatment had an increased risk of changing the therapy by the 4-year follow-up.

At the end of the follow-up period, 29 patients (52.7%) in the ON group were on second-line Disease Modifying Therapies (DMTs), compared to 14 patients (48.2%) in the OCN group (OR: 1.1; CI95%: 0.47–2.80; *p* = 0.70; *p* > 0.05), with no statistically significant difference. Additionally, 24 patients (43.6%) in the ON group and 8 patients (27.5%) in the OCN group transitioned from first-line to second-line DMTs, indicating that patients in the ON group had an increased risk of treatment change (OR: 2.06; 95% CI: 0.78–5.42; *p* = 0.03; *p* < 0.05), with a statistically significant *p*-value (Table 2; Figures 6A,B).

**Table 2:** DMT used in the two groups (ON and OCN).

	Patients	Age	Treatment at the end of follow-up (*n* of patients)
Optic nerve (ON)	55 (45 women; 10 men)	*31 ± 9.5*	*Fingomilod* (16)
			**Dimethyl Fumarate (9)**
			**IFN-β1 (5)**
			**Teriflunomide (9)**
			*Natalizumab* (6)
			*Alemtuzumab* (2)
			*Ocrelizumab* (2)
			*Cladribine* (4)
			*Siponimod* (1)
			**Glatiramer Acetate (1)**
Other cranial nerve (OCN)	29 (19 women; 10 men)	36 ± 11.6	*Fingolimod* (7)
			**Dimethyl Fumarate (6)**
			**Teriflunomide (5)**
			*Natalizumab* (5)
			*Alemtuzumab* (1)
			*Cladribine* (2)
			**Glatiramer Acetate (2)**
			*Ocrelizumab* (1)

Bolded first line treatments. Italic second line treatments.

Seven patients (12.3%) in the ON groups and 8 patients (27.5) in the OCN group started second-line treatment from the outset. Table 3 provides details about the treatments used at baseline and at the end of the follow-up, including information on both vertical and horizontal shifts.

**Table 3:** Details about the treatments among the two groups.

Group	Patients	Treatment (*n* of patients and percentage)		Shift (*n* of patients and percentage)	
		Baseline	4-years	Horizontal	Vertical
ON	55	Second line treatment (7; 12.7%)	Second Line (31; 56.4%)	14 (58.3%)	24 (50%)
		First Line treatment (48; 87.3%)	First Line (24; 43.6%)		
OCN	29	Second line treatment (8; 38%)	Second Line (16; 55.2%)	6 (28.6%)	8 (38%)
		First Line treatment (21; 62%)	First Line (13; 44.8%)		

The multiple linear regression analysis identified a weak effect of the variables examined (gender, age, nerve involved, brain lesions, and treatment used) on relapse rates, but this effect was not statistically significant (*p* = 0.09; *p* > 0.05). This is likely due to the small sample size of the analyzed patients. Cox Proportional Hazards model showed the following odds ratio: 1.03 (CI95%: 0.97–1.1) for months of relapse, 1.3 (CI95%: 0.14–10.14) for treatment used, 0.6 (CI95%: 0.09–2.91) for sex, 1.73 (CI95%: 0.33–8.52) for MRI lesions and 1.05 (CI95%: 0.97–1.13) for age in years.

## Discussion

We explored the clinical and radiological outcomes of patients with MS who presented with either optic nerve (ON) or other cranial nerve (OCN) involvement over a 4-year follow-up period. Overall, patients with MS presenting with isolated ON at onset were younger than those with OCN involvement, had a higher risk of relapsing, and experienced a greater level of disability, as measured by EDSS, throughout the 4-year follow-up period.

On the other hand, there was no significant difference in the risk of developing new radiological lesions (OR: 1.1) between the two groups, nor in the likelihood of being on second-line disease-modifying treatment by the end of the follow-up period (OR: 1.1). However, patients with ON were at a higher risk of transitioning from first-line to second-line treatment compared to those with OCN involvement at the 4-year timepoint (OR: 2.06).

Overall, despite the increased risk, the odds ratios (ORs) were rarely associated with statistically significant *p*-values. The only statistically significant value was found for the number of relapses, supporting the hypothesis that ON onset exposes patients to a higher risk of relapses. In our opinion, the lack of statistically significant results may be due to the small sample size of the two groups.

The results of the multivariate analysis indicated that all included parameters (gender, age, nerve involvement, number of new lesions, and treatment used at baseline) had a weak impact on relapses. It is important to note that both the ON group and the OCN group had patients who started since the beginning of the therapy a second line treatment, which are recommended for better control of relapse events in MS (16). However, patients in the ON group experienced more relapses and disease progression compared to those in the OCN group. If we consider the percentages (12% in the ON group and 27% in the OCN group) instead of raw numbers, this may suggest that second-line treatment could have had a positive effect on outcomes. However, this interpretation is flawed due to the retrospective nature of the study. Further prospective studies are needed to assess whether second-line treatments genuinely impact outcomes, or if OCN onset could be indicative of a less aggressive form of MS.

The frequency of second-line treatment at the end of the 4-year follow-up was similar between the two groups, although more treatment shifts occurred in the ON group. Considering the percentage of spinal lesions in the two groups 3.6% vs. 3.4% the vertical shifts should be homogeneous between the two groups. However, considering the exact number, 2 patients in ON versus 1 in the OCN group had spinal lesions during the 4 years follow-up. MS guideline suggests the use of II line treatment since the beginning (16) to contain number of relapse; because in ON there were more patients with spinal lesions and mostly of them started with a I line treatment, the higher number of vertical shifts was on line with the suggestions of the MS guideline. At the beginning of the study, we were not aware about the severity of the MS progression based on symptom-onset, considering these results, we speculate that as well as the spinal cord onset in MS, which leads to a more aggressive disease course compared to brainstem onset (1, 2, 4, 17–19), patients with ON onset might be equally considered and treated since the beginning with II line treatment (16). So, our data, despite preliminary, further extend these observations by suggesting that optic nerve involvement at onset is associated with a worse prognosis when compared to isolated involvement of other cranial nerves. Considering the optic nerves as an extension of the brain, our findings align with previous studies comparing outcomes between spinal cord and brain onset (2, 4, 17–19).

The potential relationship between spinal cord and optic nerve inflammation was first proposed in (9), suggesting that inflammation could spread from the spinal cord through the meninges to the optic nerve (9). This proposed link between the optic nerve and spinal cord was based on clinical observations, which have since been confirmed both clinically and radiologically in patients with MS (19). However, the theory that inflammation spreads rostrally from the spinal cord (9) would imply that all cranial nerves should have an equal chance of being affected. Instead, given the differential clinical outcomes observed between patients with optic nerve involvement at disease onset and those with other cranial nerve involvement (such as in the brainstem), we speculate that this may be due to the unique properties of the optic nerve.

As a CNS structure, the optic nerve is the only cranial nerve myelinated by oligodendrocytes, whereas the other cranial nerves are primarily myelinated by Schwann cells (8, 20–23). Like the brain and spinal cord (20, 21), the optic nerve is enclosed by three meningeal layers. Consequently, inflammation from either the spinal cord or brain (18) may propagate through these meningeal layers and spread to the cranial nerves, including the optic nerve. However, it remains unclear why the central myelin of the optic nerve is more susceptible to inflammation compared to the peripheral myelin (22, 24) of the other cranial nerves, and whether this contributes to the observed differences in clinical outcomes.

Our findings of a worse prognosis in ON patients compared to OCN patients differs from the results of another study, where poor prognostic factors included young age and cranial nerve inflammation (21). However, some methodological differences and different patient populations may explain these differences. First, the previous study analyzed the prevalence of cranial nerve involvement across all types of MS (Relapsing Remitting – RR, Primary Progressive – PP, and Secondary Progressive – SP), whereas we focused exclusively on RR. Secondly, Haider and colleagues grouped oculomotor, trigeminal, abducens, facial, vestibulocochlear, and vagal nerves together (excluding optic nerve involvement), rather than comparing optic nerve involvement with that of the other cranial nerves.

There may be additional anatomical explanations for the differences in reported outcomes between our study and others. The cranial nerves are myelinated for 2.6 mm at the roof entry zone (REZ) by oligodendrocytes, after which Schwann cells become responsible of myelin production (25). Given the varying susceptibility of myelin to inflammation (22), we speculate that radiological enhancement at the REZ may be associated with more aggressive inflammation targeting the myelin produced by oligodendrocytes, whereas enhancement in the peripheral portion of the nerve(s), where myelin is produced by Schwann cells, could be linked to a milder form of disease (26).

Additionally, Foesleitner et al. demonstrated through MRI neurography that microstructural changes in peripheral nerves can be observed in early MS (27). Considering OCN involvement as an early sign of the disease, such as in case of auditory symptoms (10), our findings, although based solely on clinical signs, may overlap with what is observed in MRI studies (27).

Larger studies may be necessary to further explore the neuro-inflammatory differences between the optic nerve and other cranial nerves (particularly those occurring in the REZ versus the peripheral nerve). Moreover, animal studies using valid MS models or postmortem studies may help reveal microstructural myelin differences in ON and OCN and different effect in MS prognosis.

We investigated the spread of cranial nerve involvement in the OCN subgroup. We found that the VI (abducens) nerve had the highest incidence (75%), followed by the V (trigeminal) nerve (36.7%). Only two patients presented with facial nerve palsy (VII) (8). One patient presented with hearing loss. Involvement of VIII nerve (vestibulocochlear) was far less common at onset in this cohort than the other cranial nerves. Nevertheless, auditory manifestations are well-recognized symptoms in MS. A recent systematic review, which included 1,533 MS patients, found that over time, sensorineural hearing loss occurred in approximately 25% of patients (7), although not necessarily as a presenting symptom. Corresponding lesions are predominantly localized to the medullary tegmentum in the early stages of MS (8). Cruz et al. identified hearing loss as the presenting symptom of MS in one patient in a case series of 4 patients with auditory symptoms. We observed only one case of VIII nerve involvement among 29 patients (3.4%), which seems inconsistent with the reported incidence (18). It should be noted that patients with audiovestibular symptoms are not commonly suspected to have MS and may not be initially referred to neurologists (10). Additionally, it is important to emphasize that in the early phases of MS, hearing thresholds -the standard test for hearing clinically – can be normal even in the presence of damage to the auditory pathways (28), particularly when the central pathways, rather than the cochlea or the nerve, are affected (29, 30). Therefore, auditory (and vestibular) symptoms may be under-reported in MS.

Patients in the OCN group and the one in the ON groups presented not very different percentage of new enhancing lesions, respectively 44.8% and 49%. However, the total number of lesions was greater in the ON group compared to the OCN group. It is important to note that the presence of enhancing lesions during the MRI follow-up could have been a chance occurrence.

From a clinical perspective, the results of this study may suggest the need for a more aggressive treatment approach in MS patient whose initial presentation is ON, compared to those with other cranial nerve involvement. However, this study was conducted in a small sample, and our findings should be considered preliminary. Therefore, treatment recommendations should not be based solely on cranial nerve involvement but should consider the overall clinical picture. Additionally, maintaining a high clinical suspicion for patients presenting with less typical cranial nerve involvement (e.g., facial nerve palsy, vertigo, or tinnitus) could aid in earlier diagnosis and, consequently, the early initiation of disease-modifying therapies.

### Study limitations

This study has several limitations. One of the major limitations is the small sample size (<100 participants), which means that the results should be considered preliminary. Additional studies with larger samples are necessary to validate these findings and suggest the best approach to use in patients with MS.

Secondly, the study focused on clinical and imaging data, but we did not include other important markers of neuroinflammation, such as cerebrospinal fluid oligoclonal bands, which may be relevant to MS diagnosis. Additionally, we did not calculate lesion load at baseline, which could provide valuable insight into disease progression.

Furthermore, the OCN and ON groups were not matched in size, although we believe this reflects the natural history of the condition, as optic neuritis is more common. We also did not calculate the annual relapse rate, only considering the EDSS at baseline and at the end of follow-up, without confirming disability worsening over 6 months. The absence of these measures may make it difficult to directly compare our findings with other studies.

## Conclusion

This comparative study found that patients who presented with optic nerve symptoms at the onset of MS were younger and more prone to relapses and disease progression compared to those with involvement of other cranial nerves at onset. Patients in the OCN group had better long-term outcomes, despite being older, than those with optic nerve involvement at onset. Disease outcomes may reflect differences in nerve myelination, as the optic nerve is an extension of the brain, while other cranial nerves share properties with the peripheral nervous system. Our study appears to identify a more aggressive disease course in patients with optic nerve symptoms at onset, as compared to those with involvement of other cranial nerves. However, treatment decisions should not be based solely on this factor, but should also take into account clinical presentation, serum and cerebrospinal fluid biomarkers, and MRI prognostic factors.

## Data Availability

The anonymized data supporting the conclusions of this article will be made available from the corresponding author, upon reasonable request.
